# Editorial: Enhancing public health workforce competencies: AI integration and post-pandemic educational reforms

**DOI:** 10.3389/fpubh.2026.1808782

**Published:** 2026-03-05

**Authors:** Zhaohui Su, Jianlin Jiang, Jing-Bao Nie, Kathleen Mary Gray, Dean McDonnell, Claudimar Pereira da Veiga, Yu-Tao Xiang

**Affiliations:** 1 School of Public Health, Southeast University, Nanjing, China; 2 Key Laboratory of Environmental Medicine Engineering, Ministry of Education, School of Public Health, Southeast University, Nanjing, China; 3 School of Public Health, Southeast University, Nanjing, China; 4 Department of Bioethics, Faculty of Medicine, University of Otago, Dunedin, New Zealand; 5 Centre for Digital Transformation of Health, The University of Melbourne, Parkville, VIC, Australia; 6 Department of Humanities, South East Technological University, Carlow, Ireland; 7 Fundação Dom Cabral - FDC, Nova Lima, MG, Brazil; 8 Unit of Psychiatry, Department of Public Health and Medicinal Administration, Institute of Translational Medicine, Faculty of Health Sciences, Centre for Cognitive and Brain Sciences, Institute of Advanced Studies in Humanities and Social Sciences, University of Macau, Taipa, Macao SAR, China

**Keywords:** artificial intelligence, ethics, professional development, public health, technological innovation

Public health workforce matters. The COVID-19 pandemic highlighted the critical need for adaptability and resilience within healthcare systems worldwide. The healthcare workforce, especially public health professionals, found themselves on the frontlines of a global health crisis, often ill-prepared for the unprecedented challenges posed by the pandemic. In response, medical education systems are undergoing significant transformations to address these gaps. This editorial, which addresses the research published in the Research Topic, entitled “*Enhancing public health workforce competencies: AI integration and post-pandemic educational reforms*,” aims to explore the progress, promises, and perils associated with these educational reforms, particularly through the integration of artificial intelligence (AI) and the modernization of training methodologies in healthcare systems.

## Progress: advancing healthcare education and workforce development

The pandemic created an urgent need to enhance the competencies of public health and medical professionals. Various innovative approaches have emerged to fill the gaps in healthcare education, focusing on interactive, technology-driven learning experiences that improve both knowledge retention and practical skills.

## Innovative educational models

Traditional educational frameworks have been augmented with more dynamic, student-centered teaching methodologies. One example offered by Gao et al. is the Symptom-Oriented Mind Mapping combined with Problem-Based Learning (SOM-PBL), which has been shown to improve clinical decision-making and knowledge integration in ICU clerkships. In this approach, medical students engage in more hands-on, problem-solving scenarios that foster deeper engagement with the material and enhance critical thinking. Similarly, the integration of Evidence-Based Medicine (EBM) within the BOPPPS model has demonstrated improved clinical competence and confidence among neurology clerkships, as indicated in the research conducted by Chen et al. This method not only enhances students' knowledge but also boosts their ability to evaluate clinical questions critically—skills that are indispensable in both primary care and specialized settings.

## Simulation and technology in healthcare training

Simulation-based education has proved invaluable during the pandemic, particularly for practicing complex clinical skills in a controlled environment. A systematic review on simulation-based airway management during the COVID-19 pandemic, conducted by Kohler et al., confirmed that such training significantly enhanced healthcare providers' confidence and preparedness for handling infectious patients. Simulation technologies allow for repeated practice in high-stakes situations without putting patients at risk, which is essential during a health crisis where rapid, decisive action is needed. These findings suggest that simulation should be a permanent component of medical and public health education going forward.

## Capacity building in low-resource settings

While high-income countries have focused on adopting high-tech solutions, low-resource settings have also made significant strides in workforce development, especially in public health. For instance, as investigation conducted by Nguinkal et al. showed, a capacity-strengthening model across East Africa trained public health professionals in pathogen genomics, antimicrobial resistance (AMR) analysis, and bioinformatics. This initiative underscored the importance of One Health frameworks in strengthening national health systems. It provides a model for future workforce training programs in low-income areas, ensuring that even countries with limited resources can be prepared for the next pandemic or health emergency.

## Promises: the potential of AI and technological advancements in public health education

AI is increasingly recognized as a transformative force in education and healthcare. The integration of AI-driven tools into medical education and workforce development presents promising opportunities to enhance learning experiences, optimize clinical decision-making, and address inefficiencies in healthcare systems.

## AI-driven personalized learning

One of the most promising uses of AI in healthcare education is the development of personalized learning platforms. A study by Chen evaluating the impact of AI-driven learning platforms on medical students found that AI significantly enhanced students' academic performance, self-directed learning behaviors, and engagement. These platforms use real-time data analytics to adapt the learning path to each student's needs, ensuring a more individualized learning experience. Furthermore, these systems provide tailored feedback, which helps students remain engaged and progress at their own pace. Such technology holds great potential for optimizing educational outcomes, especially in diverse learning environments.

## AI for pandemic preparedness

AI's role in public health education extends beyond classroom learning. AI-driven epidemiological modeling has the potential to revolutionize pandemic preparedness by predicting outbreaks, analyzing disease transmission patterns, and optimizing resource allocation. The study conducted by Abdelouahed et al. on AI in public health education emphasizes the value of AI in creating predictive models that can forecast the trajectory of infectious diseases like COVID-19 and monkeypox, thus enhancing both individual and collective response capabilities. By integrating AI into public health education programs, students and professionals can gain a deeper understanding of how AI can be leveraged for more effective outbreak management. Systematic change and optimization are also vital. Take, for example, the nationwide population-based study evaluated trends in surgical and anesthetic practices before and during the COVID-19 pandemic in South Korea. Ahn et al. showed that, while overall surgical volume remained relatively stable, practice patterns shifted, with increased use of regional anesthesia and monitored anesthesia care. These findings suggest adaptive changes in perioperative management and highlight the importance of strategic planning to maintain surgical services during public health crises.

## Educational competitions and public health engagement

Another area where AI can enhance public health education is through academic competitions. These competitions, which focus on real-world public health issues, as indicated by Zhou et al., foster important skills like teamwork, problem-solving, and communication. In the context of AI, such competitions could be augmented with data analytics tools, allowing students to engage with real-time data and practice decision-making in simulated public health crises. This integration of AI into public health competitions could further deepen students' skills in analyzing public health challenges and formulating evidence-based solutions.

## Hybrid learning and resilience

The pandemic highlighted the need for more flexible educational models that balance digital learning with traditional in-person instruction. The transition to hybrid learning environments during the COVID-19 crisis has been a double-edged sword. While it posed challenges related to digital access and student isolation, it also demonstrated the value of flexible, hybrid learning models, as research by Schwartz et al. highlighted. Students reported greater convenience in accessing materials remotely, but many also expressed a need for more in-person interactions to combat the sense of isolation. The future of medical education lies in finding the right balance between digital flexibility and structured in-person engagement, with AI tools playing a key role in ensuring students remain connected and engaged.

## Perils: challenges and risks in AI integration and educational reforms

Despite the considerable promises offered by AI and other technological innovations, there are significant challenges and risks that need to be addressed. These include ethical concerns, digital equity issues, and the risk of diminishing the human element of healthcare.

## Data privacy and ethical concerns

The use of AI in educational and clinical settings raises important ethical and data privacy concerns. AI-driven systems collect vast amounts of data about students' learning behaviors and healthcare practitioners' performance. Without robust data protection policies, this could expose sensitive information to exploitation or misuse. Additionally, as findings from Chen suggested, the algorithms used to personalize learning or clinical decision-making may inadvertently reinforce biases, leading to inequities in healthcare delivery and education. There is a pressing need for the development of clear ethical guidelines to govern the use of AI in medical education and practice.

## Digital divide and inequality

As healthcare education increasingly moves online, there is a growing risk of exacerbating existing digital divides that often worsen the challenges faced by the most vulnerable communities, from low-income older adults to rural adolescents. As results from Schwartz et al. highlighted, many students and healthcare workers, particularly in low- and middle-income countries, often lack access to the necessary digital tools and Internet infrastructure to benefit from AI-driven educational platforms. This divide might be particularly more pronounced for young health professionals living in societies where cutting-edge technologies, like large language models (LLMs), are not made open or highly-priced. This digital divide, particularly in the dire contrasts between open-ended and subscription-based AI applications, could further hinder the broader implementation of AI in public health education and workforce training, leaving behind those who are already most vulnerable in global health emergencies. Ensuring equitable access to technology is critical to prevent further inequality in healthcare training and delivery.

## Over-reliance on technology

While AI has the potential to optimize decision-making, there is a danger of over-relying on technology at the expense of human expertise. The study by Alharbi on ICU nurses' self-efficacy during the COVID-19 pandemic highlights the importance of maintaining human judgment in clinical practice. While AI can assist in managing data and providing clinical recommendations, healthcare professionals must retain the ability to critically analyze situations and make informed decisions. Over-reliance on AI tools could lead to a loss of critical thinking skills among healthcare professionals, particularly in complex or ambiguous situations where human intuition is crucial.

## Workforce inequities in public health

Workforce disparities persist in the public health sector, especially in regions where infrastructure and training opportunities are limited. The study conducted by Zhang et al. on human resource inequities in China's CDCs highlighted significant disparities in workforce distribution, which compounded challenges during the pandemic. Such inequalities can hinder effective pandemic response efforts and limit the capacity for equitable healthcare delivery. AI solutions must be designed to address these inequities by providing targeted support and resources, as Akingbola et al. highlighted in their study, from kindred international collaborations to cultural-intelligent local innovations—to underserved regions, rather than exacerbating existing gaps.

## Ethics and human values

Advances in science, technology, and medicine always raise moral questions. So, it is indispensable that enhancing public health workforce competencies through AI integration and educational reforms incorporates ethical competency. Ethical competency should include knowledge of ethical principles and norms, the skills to employ them effectively, and moral imagination in healthcare practices. As already mentioned, several articles in this collection have addressed ethical issues, particularly the persistent problems of inequality and privacy protection, the challenge of risk management, and the inherent threats posed by technologies to human values. Yet, among others, an area for more specific and in-depth future exploration is how to uphold human values rooted in Western and, especially, non-Western cultures to safeguard the adoption of technologies that serve the welfare of patients and healthcare professionals. For example, such fundamental traditional Confucian ethical ideals and principles as ren (humaneness or humanity), yi (righteousness, rightness, or justice), li (ritual, rule-abiding), and xin (trust, trustworthiness, faithfulness) are not just relevant but essential to navigate morally sound frameworks and mechanisms in the efforts to enhance health workforce competencies in the age of AI, not only in China and East Asia but globally.

## Conclusion

As we look ahead, the future of public health education and workforce development lies in effectively integrating AI and other digital technologies into training systems. The progress made in developing personalized learning models, simulation-based education, and capacity-building programs provides a solid foundation for enhancing public health competencies globally. However, it is essential to carefully navigate the challenges of data privacy, digital equity, and ethical concerns to ensure that the benefits of AI are equitably distributed. By addressing these risks, we can ensure that AI and technological innovations serve to enhance the effectiveness and resilience of the healthcare workforce, preparing them for the next global health crisis.

